# Activity, Template Preference, and Compatibility of Components of RNA Replicase of Eastern Equine Encephalitis Virus

**DOI:** 10.1128/jvi.01368-22

**Published:** 2022-12-19

**Authors:** Laura Sandra Lello, Agneta Miilimäe, Liubov Cherkashchenko, Ailar Omler, Rachel Skilton, Rachel Ireland, David Ulaeto, Andres Merits

**Affiliations:** a Institute of Technology, University of Tartu, Tartu, Estonia; b CBR Division, Dstl Porton Down, Salisbury, UK; Loyola University Chicago

**Keywords:** EEEV, RNA replicase, RNA replication, alpavirus, template RNA

## Abstract

Eastern equine encephalitis virus (EEEV) usually cycles between Culiseta melanura mosquitoes and birds; however, it can also infect humans. EEEV has a positive-sense RNA genome that, in infected cells, serves as an mRNA for the P1234 polyprotein. P1234 undergoes a series of precise cleavage events producing four nonstructural proteins (nsP1–4) representing subunits of the RNA replicase. Here, we report the construction and properties of a *trans*-replicase for EEEV. The template RNA of EEEV was shown to be replicated by replicases of diverse alphaviruses. The EEEV replicase, on the other hand, demonstrated limited ability in replicating template RNAs originating from alphaviruses of the Semliki Forest virus complex. The replicase of EEEV was also successfully reconstructed from P123 and nsP4 components. The ability of EEEV P123 to form functional RNA replicases with heterologous nsP4s was more efficient using EEEV template RNA than heterologous alphavirus template RNA. This finding indicates that unlike with previously studied Semliki Forest complex alphaviruses, P123 and/or its processing products have a leading role in EEEV template RNA recognition. Infection of HEK293T cells harboring the EEEV template RNA with EEEV or Western equine encephalitis virus prominently activated expression of a reporter encoded in the template RNA; the effect was much smaller for infection with other alphaviruses and not detectable upon flavivirus infection. At the same time, EEEV infection resulted only in a limited activation of the template RNA of chikungunya virus. Thus, cells harboring reporter-carrying template RNAs can be used as sensitive and selective biosensors for different alphaviruses.

**IMPORTANCE** Infection of EEEV in humans can cause serious neurologic disease with an approximately 30% fatality rate. Although human infections are rare, a record-breaking number was documented in 2019. The replication of EEEV has a unique requirement for host factors but is poorly studied, partly because the virus requires biosafety level 3 facilities which can limit the scope of experiments; at the same time, these studies are crucial for developing antiviral approaches. The EEEV *trans*-replicase developed here contributes significantly to research on EEEV, providing a safe and versatile tool for studying the virus RNA replication. Using this system, the compatibility of EEEV replicase components with counterparts from other alphaviruses was analyzed. The obtained data can be used to develop unique biosensors that provide alternative methods for detection, identification, quantitation, and neutralization of viable alphaviruses that are compatible with high throughput, semiautomated approaches.

## INTRODUCTION

Alphaviruses (family *Togaviridae*) are emerging human and animal pathogens. Most known alphaviruses are arboviruses (1) although some alphaviruses such as Eilat virus (EILV) lack a known vertebrate host, infecting only insects (2). Alphaviruses are often divided into Old World and New World viruses (3). Old World alphaviruses are associated with an arthritogenic phenotype and include the medically important chikungunya virus (CHIKV) as well as the extensively studied model viruses, Semliki Forest virus (SFV) and Sindbis virus (SINV). New World alphaviruses, on the other hand, are encephalitic and include Venezuelan equine encephalitis virus (VEEV), Western equine encephalitis virus (WEEV) and Eastern equine encephalitis virus (EEEV). Each group can be further divided into complexes based on sequence similarities and antigenic properties. The latest report of the International Committee on the Taxonomy of Viruses (ICTV) recognizes Venezuelan equine encephalitis complex, Eastern equine encephalitis complex, Western equine encephalitis complex (including also SINV), Barmah Forest complex with Barmah Forest virus (BFV) as the sole member, Middelburg complex, Ndumu complex and Semliki Forest complex that includes, among others, SFV, CHIKV, o’nyong’nyong virus (ONNV), Mayaro virus (MAYV), and Ross River virus (RRV) (3).

EEEV is considered one of the most pathogenic alphaviruses. It is enzootic in the northeastern United States, cycling between Culiseta melanura mosquitoes and birds in forested swamp areas (4, 5). EEEV infection in humans most commonly results in febrile illness with a high chance of complete recovery. However, infected people can develop neurological diseases like meningitis or encephalitis that can be fatal (6) and often result in lifelong neurological impairments in survivors (5).

Alphaviruses have approximately 12-kb long positive-sense RNA genomes equipped with a cap0 structure in the 5′ terminus and a poly(A) tail in the 3′ terminus (7). The genome comprises two open-reading frames (ORFs) and three untranslated regions (UTR): 5′ UTR, intergenic region, and 3′ UTR; these regions contain *cis*-elements important for viral RNA synthesis. ORF2 encodes the structural proteins that are translated from the subgenomic (SG) mRNA synthesized in infected cells (8). ORF1 is translated from the genomic RNA and encodes the P1234 polyprotein, the precursor of alphavirus replicase proteins (nsP1–4) (9). nsP1 is the viral capping enzyme and has the ability to interact with membranes, acting as an anchor for the replication complexes (RC) (10, 11). nsP2 is an RNA helicase, nucleoside triphosphatase (NTPase), and RNA triphosphatase and has proteolytic activity necessary for processing P1234 (12). It has also been shown that nsP2 can bind RNA and, via this, take part in RNA replication (13, 14). nsP4 is present in lower quantities relative to the other nsPs in infected cells due to a leaky opal stop codon at the end of the nsP3 region and a destabilizing Tyr residue at the N terminus of nsP4 (15). The C-terminal region of nsP4 comprises the RNA-dependent RNA polymerase (RdRp) domain and contains the conserved catalytic GDD motif characteristic to RdRps (16, 17). Twelve molecules of nsP1, one molecule of nsP2, and one molecule of nsP4 form the core of the RNA replicase. nsP3 is not a part of this complex; instead, it forms a cytoplasmic ring structure associated with active RCs (18). nsP3 consists of three domains: the N-terminal macrodomain, the central domain unique to alphaviruses, and the C-terminal hypervariable domain (HVD). HVD is intrinsically disordered and acts as an assembly hub for different host factors required for RNA replication, including FXR and G3BP protein family members (19). Old World alphaviruses, including BFV, RRV, MAYV, ONNV, and CHIKV are unable to effectively replicate RNA in G3BP1/2 knockout cells, while VEEV exhibits high RNA replication (20). This suggests that Old World alphaviruses require G3BP proteins for effective replication; in contrast, the New World alphaviruses are dependent on the FXR protein family members (21). As an exception, HVD of EEEV interacts with members of both FXR and G3BP protein families (22).

Alphavirus RCs are located at the necks of membranous invaginations that are often called the spherules (12). The formation of RCs requires the presence of P1234, its processing intermediates, mature nsPs, genomic RNA, and host factors. RC formation and processing of P1234 are coupled. Firstly, nsP4 is cleaved from P1234 and the P123 + nsP4 complex, named an early replicase and capable of synthesizing negative-strand RNA, is formed. Next, nsP1 is cleaved from P123 resulting in the formation of a short-lived nsP1 + P23 + nsP4 replicase. The final cleavage of P23 leads to the formation of the stable late replicase that synthesizes positive-strand RNAs (23). Alphavirus RNA replication and RC formation can be modeled by coexpressing a replication-competent template RNA and P1234, P123 + nsP4, or nsP1 + uncleavable P23 + nsP4 (24). Such systems, often called *trans*-replicases, represent valuable tools to study alphavirus RNA replication. Uncoupling RNA replication from the expression of viral replicase proteins/their precursors simplifies the analysis of the impact of different mutations on RNA replication. In addition, *trans*-replicases allow analysis of the compatibility of components of RNA replicases originating from different alphaviruses. For these reasons, we have previously developed *trans*-replicases for several viruses from the SFV complex (CHIKV, ONNV, RRV, SFV, and MAYV) as well as for SINV, BFV, EILV, and VEEV. These systems have been used for analyzing the cross-utilization of template RNAs (25) and for reconstructing RNA replicases from P123 and nsP4 components belonging to different alphaviruses (26), providing novel insights into the roles of different components in RC formation.

EEEV belongs to the Eastern equine encephalitis complex, the only major complex of alphaviruses not included in our previous studies (20, 25, 26). The molecular mechanisms of EEEV RNA replication are rather poorly understood, partly because of the requirement of biosafety level 3 containment. Here, we report properties unique for EEEV RNA replicase and properties shared with replicases of other alphaviruses, revealed using an EEEV *trans*-replicase system. A novel method for virus detection and identification, and enumeration of unmodified viruses applicable for various high-throughput assays, was developed. Combined, our data contributes to understanding the RNA replication of EEEV and alphaviruses in general. It also offers a versatile and effective way to study the replication of EEEV and other alphaviruses in the future.

## RESULTS

### The *trans*-replicase of EEEV is highly active.

**FIG 1:**
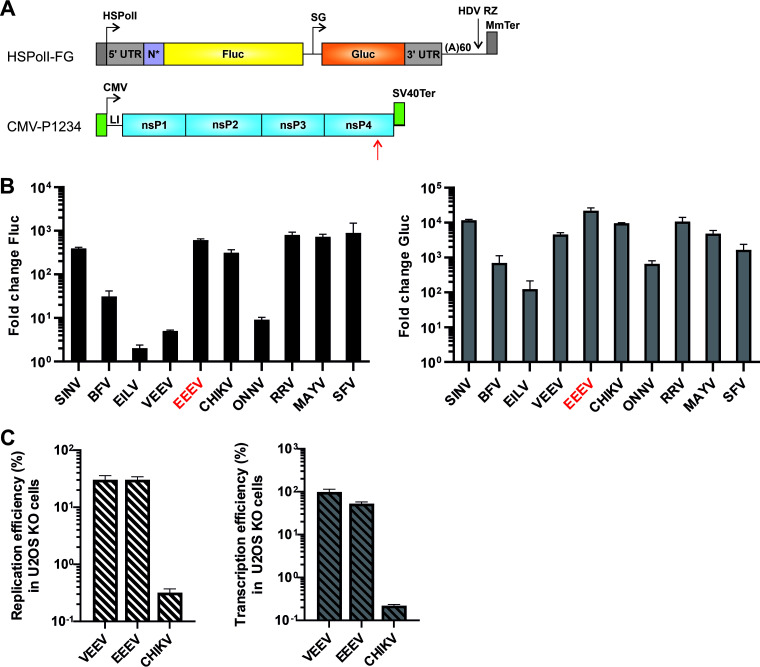
The *trans*-replicase of EEEV is highly active in human cells. (A) Schematic presentation of the template RNA (top) and the P1234 expression constructs (bottom). HSPolI, a truncated promoter (residues −211 to −1) for human RNA polymerase I; 5′ UTR, full-length 5′ UTR of an alphavirus; nsP1 N*, region encoding the N-terminal 77 to 114 amino acid residues of nsP1, depending on the virus; SG, SG promoter spanning (with respect to the termination codon of nsP4) from position −79 to the end of the intergenic region; 3′ UTR, truncated (last 110 residues) 3′ UTR of an alphavirus; HDV RZ, antisense strand ribozyme of hepatitis delta virus; MmTer, a terminator for RNA polymerase I in mice; CMV, immediate early promoter of human cytomegalovirus; LI, leader sequence of the herpes simplex virus thymidine kinase gene with artificial intron; SV40Ter, simian virus 40 late polyadenylation region. The red arrow indicates the location of the GDD motif in nsP4; in polymerase-negative constructs, this was replaced by GAA. The vector backbones are not shown, and drawings are not to scale. (B) HEK293T cells in 96-well plates were cotransfected with matching pairs of CMV-P1234 and HSPolI-FG plasmids. As the negative control, CMV-P1234^GAA^ was used instead of CMV-P1234. Cells were incubated at 37°C and lysed at 18 h posttransfection (hpt); cells transfected with plasmids containing sequences from EILV were incubated at 28°C and lysed at 48 hpt. Fluc (marker of replication) and Gluc (marker of transcription) activities produced by active replicases were normalized to the P1234^GAA^ controls. The value obtained for the P1234^GAA^ controls was taken as 1. The means ± standard deviation (SD) of three independent experiments are shown. (C) U2OS and U2OS ΔΔ cells grown on 12-well plates were cotransfected with matching combinations of the template RNA and replicase proteins expression plasmids of VEEV, EEEV, and CHIKV. Transfected cells were incubated at 37°C for 18 h, lysed, and Fluc and Gluc activities were measured. Data represents the ratios of replication and transcription signals in U2OS ΔΔ cells to wild-type U2OS cells (taken as 100%). The *x* axis represents different *trans*-replicases. The means ± SD of three independent experiments are shown.

The *trans*-replication assay presents an effective way of studying alphavirus RNA replication in the absence of reversions and compensatory changes (27). Importantly, this system eliminates the need for using high biological safety level facilities required for studying highly pathogenic alphaviruses, or construction of recombinant/chimeric viruses. Previous studies have shown that *trans*-replicases represent sensitive, reliable, and biologically relevant research tools (14, 20, 25, 26, 28, 29). As EEEV is highly pathogenic but poorly studied, we designed and constructed a corresponding *trans*-replication system for EEEV using the same approach previously used for other alphaviruses (25) (Fig. 1A). As in previous studies (25, 26, 30), activity of RNA replicases were mostly analyzed using markers incorporated into the replicating template RNA: firefly luciferase (Fluc) under the genomic promoter and *Gaussia* luciferase (Gluc) under the SG promoter (Fig. 1A). For simplicity, hereafter, the full-length RNA serving as a template for Fluc expression is termed “genomic RNA” (and its synthesis as “replication”), the RNA synthesized from the SG promoter and serving as a template for Gluc expression is termed “SG RNA” (and its synthesis as “transcription”), and all RNAs synthesized by *trans*-replicases are referred to as “viral RNAs”. The efficiencies of replication and transcription were estimated by fold changes (“boost”) of the corresponding reporter expression in cells expressing active replicase proteins compared to cells expressing P1234 harboring a mutation in the active site of nsP4 (P1234^GAA^), as previously described (30). Combinations of plasmids expressing proteins and template RNAs from the same alphavirus are referred to as “matching combinations” while the combinations expressing components from different viruses are referred to as “heterologous combinations.”

The *trans*-replicases of the majority of arthropod-transmitted alphaviruses are highly active in mammalian (including human) cells; however, the replication and transcription activities of the *trans*-replicases of BFV and ONNV are relatively low, as is the replication activity of VEEV *trans*-replicase (25, 27, 28, 30). Here, the EEEV *trans*-replicase was found to be highly active in human cells. A comparison with previously constructed *trans*-replicases revealed that the replication activity of EEEV *trans*-replicase was at a similar level with those of SINV, CHIKV, RRV, MAYV, and SFV. Interestingly, the replication activity of EEEV *trans*-replicase hugely (approximately 120-fold, *P* < 0.0001) exceeded that of the *trans*-replicase of VEEV, another New World alphavirus (Fig. 1B). For transcription, the activity of the *trans*-replicase of EEEV exceeded those of all other *trans*-replicases (Fig. 1B).

It has been shown that the replication of the New World and Old World alphaviruses is dependent on host proteins from different protein families (20, 21). Knock-out of the G3BP1/2 proteins strongly reduces the activities of the *trans*-replicase of CHIKV but has no impact on the activities of the *trans*-replicase of VEEV, which binds the FXR proteins (20). EEEV is the only alphavirus whose nsP3 has been shown to bind and use members of both the G3BP and FXR protein families (21, 22). To analyze the impact of the knockout of G3BP1/2 proteins on the activities of the EEEV *trans*-replicase, we compared its activities in U2OS ΔΔ (G3BP1/2 knockout) cells with its activities in wild type U2OS cells. In accordance with a previous study (20), the lack of G3BP1/2 proteins had only a minor effect on the activities of the VEEV *trans*-replicase. In contrast, activities of the CHIKV *trans*-replicase were drastically reduced (Fig. 1C). Like the *trans*-replicase of VEEV, only a minor impact of the absence of G3BP1/2 proteins was observed on the activities of the *trans*-replicase of EEEV (Fig. 1C). This finding is in accord with a previous study reporting that the lack of interaction with either FXR or G3BP family proteins has no effect on EEEV replication (22).

### EEEV template RNA is promiscuous, while its replicase proteins use the template RNAs of viruses from the SFV complex poorly.

**FIG 2:**
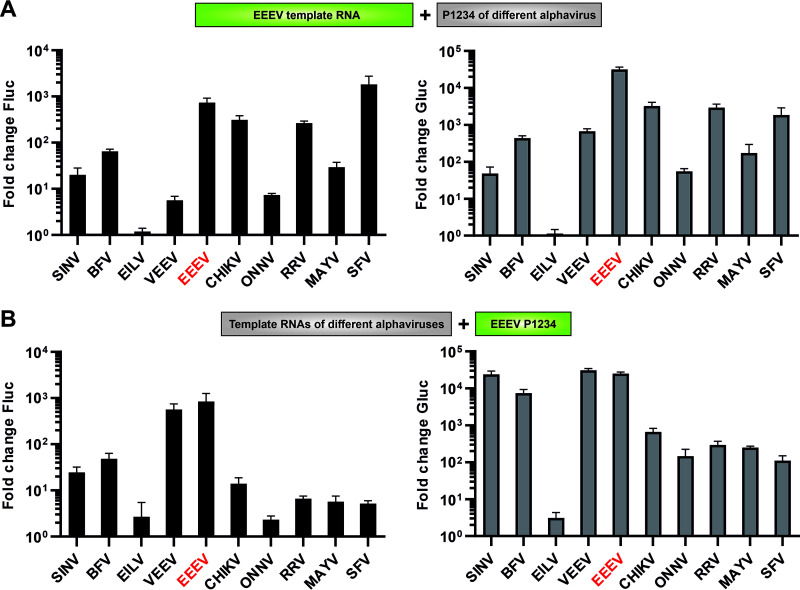
Cross-utilization of the template RNA and the replicase proteins of EEEV are similar to that of outgroup alphaviruses. (A) HEK293T cells in 96-well plates were cotransfected with HSPolI-FG-EEEV and CMV-P1234 or CMV-P1234^GAA^ from an alphavirus shown at horizontal axes. Transfected cells were incubated at 37°C and lysed 18 hpt; cells transfected with plasmids originating from EILV were incubated at 28°C and lysed 48 hpt. (B) HEK293T cells were cotransfected with HSPolI-FG from an alphavirus shown at horizontal axes and CMV-P1234-EEEV or CMV-P1234^GAA^-EEEV. Transfected cells were incubated at 37°C and lysed 18 hpt. (A, and B) Data represent the activities of Fluc (left panels) and Gluc (right panels) from CMV-P1234-transfected cells normalized to the paired CMV-P1234^GAA^ control cells. The value obtained for P1234^GAA^ controls was taken as 1; activities lower than that observed for P1234^GAA^ are also shown as 1. The means ± SD of three independent experiments are shown.

Alphavirus replicase proteins have been shown to cross-utilize heterologous template RNAs. The template RNAs of viruses belonging to the SFV complex are efficiently used only by the replicase proteins of viruses belonging to the same complex while the template RNAs of SINV, BFV, VEEV, and EILV (hereafter “outgroup alphaviruses”) are promiscuous (25). Analysis performed using HEK293T cells and EEEV template RNA revealed that the template RNA of EEEV was used not only by the matching replicase proteins but also by those of all the heterologous alphaviruses except for EILV (Fig. 2A). The poor replication observed in the case of the replicase proteins of VEEV and limited replication/transcription by replicase proteins of ONNV were unlikely due to the EEEV RNA template as the replicase proteins of these two viruses had relatively low activities also on their matching templates (compare Fig. 1B and Fig. 2A). Thus, the template RNA of EEEV did not exhibit specific behavior toward the replicase proteins of alphaviruses belonging to different complexes. The inability of EILV replicase to use the RNA template of EEEV (Fig. 2A) is most likely due to the use of human cells where the EILV replicase was virtually inactive on all heterologous RNA templates except for that of VEEV (25).

The cross-utilization of template RNAs by alphavirus replicase proteins is not symmetrical (25). Again, the properties of the replicase proteins of EEEV were found to be consistent with this trend. In HEK293T cells, the EEEV replicase proteins used the VEEV template RNA with almost the same efficiency as they used its matching template RNA (Fig. 2B). The only template RNA that was not used by the replicase proteins of EEEV was the template RNA of EILV (Fig. 2B), a property shared with the replicase proteins of VEEV but not with those of other alphaviruses (25). Thus, the data from these experiments is in accord with the relationship between these two New World alphaviruses. The template RNAs of SINV and BFV were replicated at moderate levels and transcribed at high levels. In contrast, both replication and transcription of the template RNAs of viruses from the SFV complex were relatively inefficient (Fig. 2B). Thus, the properties of the template RNA and the replicase proteins of EEEV are similar to those of outgroup alphaviruses and EEEV was therefore considered a member of this group.

### The *trans*-replicase of EEEV requires an optimal ratio of P123 and nsP4 components for maximal activity.

**FIG 3:**
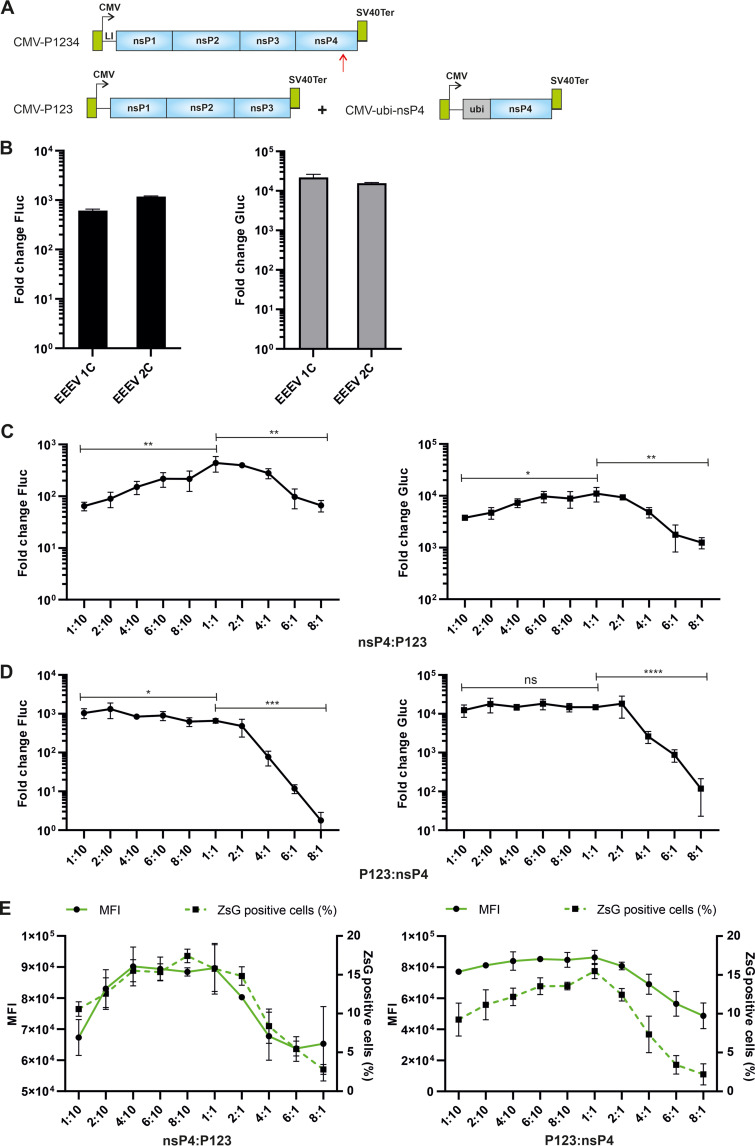
Activity of the two-component *trans-*replicase of EEEV depends on the optimal ratio of P123:nsP4 expression plasmids. (A) Schematic presentation of P1234 (top) and P123 + nsP4 (bottom) expression constructs. Ubi, sequence encoding human ubiquitin; other designations are same as in legend of Fig. 1A. The vector backbones are not shown, and drawings are not to scale. (B) HEK293T cells in 96-well plates were cotransfected with combinations of HSPolI-FG-EEEV template RNA expression plasmid and CMV-P1234-EEEV (1C) or CMV-P123-EEEV + CMV-ubi-nsP4-EEEV (2C). As negative control, CMV-P1234^GAA^-EEEV was used. (C) HEK293T cells were cotransfected with combinations of and HSPolI-FG-EEEV, CMV-P123-EEEV, and CMV-ubi-nsP4-EEEV. As a negative control, CMV-P1234^GAA^-EEEV was used. The amounts of HSPolI-FG-EEEV and CMV-P123-EEEV were kept constant, while the CMV-ubi-nsP4-EEEV was provided at a 1:10, 2:10, 4:10, 6:10, 8:10, 1:1, 2:1, 4:1, 6:1, or 8:1 molar ratio in respect to CMV-P123-EEEV. (D) The experiment was performed as described for (C) except that the amounts of HSPolI-FG-EEEV and CMV-ubi-nsP4-EEEV were kept constant, while the CMV-P123-EEEV was provided at a 1:10, 2:10, 4:10, 6:10, 8:10, 1:1, 2:1, 4:1, 6:1, or 8:1 molar ratio in respect to CMV-ubi-nsP4-EEEV. (B–D) Transfected cells were incubated at 37°C and lysed 18 hpt, data represent the activities of Fluc (left panels) and Gluc (right panels) from CMV-P1234-EEEV or CMV-P123-EEEV + CMV-ubi-nsP4-EEEV-transfected cells normalized to the paired CMV-P1234^GAA^-EEEV control cells. The value obtained for the P1234^GAA^ control was taken as 1. The means ± SD of three independent experiments are shown. ns, not significant; *, *P* < 0.05; **, *P* < 0.01; ***, *P* < 0.001; ****, *P* < 0.0001; Student’s unpaired one-tailed *t* test. (E) HEK293T cells in 24-well plates were cotransfected with combinations of HSPolI-FZsG-EEEV, CMV-P123-EEEV, and CMV-ubi-nsP4-EEEV. The amounts of HSPolI-FZsG-EEEV and CMV-P123-EEEV were kept constant, while the CMV-ubi-nsP4-EEEV was provided at a 1:10, 2:10, 4:10, 6:10, 8:10, 1:1, 2:1, 4:1, 6:1 or 8:1 molar ratio in respect to CMV-P123-EEEV (left). The amounts of HSPolI-FZsG-EEEV and CMV-ubi-nsP4-EEEV were kept constant, while the CMV-P123-EEEV was provided at a 1:10, 2:10, 4:10, 6:10, 8:10, 1:1, 2:1, 4:1, 6:1, or 8:1 molar ratio in respect to CMV-ubi-nsP4-EEEV (right). Cells were incubated at 37°C for 18 h, collected, and analyzed with an Attune NxT acoustic focusing cytometer. The percentage of ZsGreen-positive cells from living cells and mean fluorescence intensity (MFI) in arbitrary units for ZsGreen-positive cells are shown. The means ± SD of three independent experiments are shown.

The P123 and nsP4 of alphaviruses, expressed from separate expression plasmids, can form an active RNA replicase. This system, hereafter termed the “two-component replicase” (Fig. 3A), has proven to be an effective and versatile tool that has been used for studying RC formation (24) and the effects of mutations in nsP4 on the activities of RNA replicases of RRV and SINV (16). Here, we constructed the two-component *trans*-replicase of EEEV and compared its activity with the replicase expressed from the CMV-P1234-EEEV expression plasmid (hereafter “one-component replicase”). It was observed that when the plasmids expressing P123 and nsP4 components were used in 1:1 ratio, the activities of the two-component *trans*-replicase of EEEV were similar to these of one-component *trans*-replicase. Only a modest (approximately 1.9-fold) increase of replication and a minimal (approximately 1.4-fold) decrease of transcription activities were observed for two-component replicase (Fig. 3B).

The nsP4 of alphaviruses is an unstable protein and for most of the alphaviruses it is also produced in a smaller amount compared to other nsPs. Thus, the stoichiometry of the P123 (and its cleavage products) and the nsP4 in cells transfected with the two-component *trans*-replicase plasmids can impact RNA replication and transcription activities. Indeed, the activities of the two-component *trans*-replicases of CHIKV, SINV, BFV, VEEV, and EILV increase alongside the increase of the nsP4 relative to P123 expression plasmids ratio (26). Similar analysis performed with the two-component *trans*-replicase of EEEV revealed a trend partly consistent with the observation made for the *trans*-replicases of the above listed viruses. Increase of the nsP4:P123 expression plasmid ratio from 1:10 to 1:1 resulted in an approximately 7-fold increase of replication and a 3-fold increase of transcription activities of the EEEV *trans*-replicase (Fig. 3C). However, further increase of the nsP4:P123 expression plasmid ratio resulted in a significant decrease of the activities of the EEEV *trans*-replicase: at the ratio of 8:1, the activities were approximately 7- to 8-fold lower than at the 1:1 ratio (Fig. 3C). In this regard, the two-component *trans*-replicase of EEEV contrasts to those of CHIKV, SINV, BFV, VEEV, and EILV, the activities of which either continued to increase or plateaued at nsP4:P123 expression plasmid ratios higher than 1:1 (26). This finding indicates the existence of an optimal ratio of nsP4 to P123 for EEEV, exceeding which results in a reduction of the RNA replicase activities. This assumption was confirmed in an experiment where the amount of the nsP4 expression plasmid was kept constant and the amount of the P123 expression plasmid was varied. Increasing the amounts of the P123:nsP4 expression plasmids ratio from 1:10 to 1:1 had a minimal effect on the efficiency of the EEEV RNA replication and transcription (Fig. 3D). However, increasing the P123:nsP4 expression plasmid ratios from 1:1 to 8:1 had a debilitating effect on the RNA replication (approximately 370-fold decrease) and transcription (approximately 125-fold decrease) (Fig. 3D). One possible explanation for these effects is that the high amounts of nsP4 (Fig. 3C) or P123 (Fig. 3D) expression plasmids or products encoded by these plasmids were toxic to the cells. However, we did not detect cytopathic effects (CPE) by visual observation of the transfected cells, nor did we observe a decrease of the percentage of living cells using flow cytometry. Therefore, it was concluded that the formation of the RNA replicase of EEEV occurs most efficiently at a certain P123:nsP4 ratio and possibly depends also on the ratio of these components to the template RNA. In our experiments the optimal conditions were achieved when the expression plasmids of all these components were used in equimolar amounts.

The differences in the RNA replication and transcription activities may originate from a difference in the percentage of the transfected cells where RNA replication is initiated, from the efficiency of the RNA replication in these cells, or from the combination of these two factors. To differentiate between these possibilities, the plasmid for EEEV template RNA expression was modified: the sequence encoding for Gluc was substituted with that of the ZsGreen. Use of the obtained HSPolI-FZsG-EEEV plasmid revealed that increasing the nsP4:P123 expression plasmid ratio from 1:10 to 4:10 results in an increase of the percentage of ZsGreen-positive cells (i.e., cells where RNA replication was initiated) approximately 1.5-fold (*P* < 0.01). The increase of the ratio also resulted in an approximately 1.4-fold (*P* < 0.01) increase of the mean-fluorescence intensity (MFI) in the ZsGreen-positive cells (i.e., increase of transcription activity per cell with viral RNA replication) (Fig. 3E, left). Thus, the observed increase of the activity of the EEEV *trans*-replicase (Fig. 3C) was caused by a combination of these two effects. Subsequent increase of the nsP4:P123 expression plasmid ratio from 4:10 to 1:1 had no significant effect on the percentage of ZsGreen-positive cells or MFI, while ratios 1:1 and above resulted in a sharp decline of both the percentage of fluorescent cells and MFI (Fig. 3E, left). Thus, the observed decline of RNA replication and transcription at high nsP4:P123 expression plasmid ratios also resulted from a combination of the decreased numbers of cells with RNA replication and the decreased efficiency of RNA synthesis per cell. We also conducted a flow cytometry analysis of the cells transfected with a constant amount of nsP4 expression plasmid and different amounts of P123 expressing plasmid. Increase of the P123:nsP4 expression plasmid ratio from 1:10 to 1:1 increased the percentage of ZsGreen-positive cells approximately 1.7-fold but had only minimal effect on the MFI (Fig. 3E, right). However, consistent with the data obtained using the HSPolI-FG-EEEV plasmid, the increase of the P123:nsP4 expression plasmid ratio from 1:1 to 8:1 resulted in a sharp decline of both the percentage of ZsGreen-positive cells and MFI in these cells (Fig. 3E, right). Combined, these results suggest that, similarly to replicases of previously studied alphaviruses, the nsP4 also serves as a rate limiting component for the EEEV RNA replicase. However, it must be expressed in an optimal ratio to the P123 component (and possibly the template RNA), as levels of nsP4 exceeding the optimal levels have a strong negative impact on the activity of the EEEV RNA replicase.

### Activity of the three-component EEEV *trans*-replicase depends on the ratio of nsP4 to nsP1+P2^CA^3 expression plasmids.

**FIG 4:**
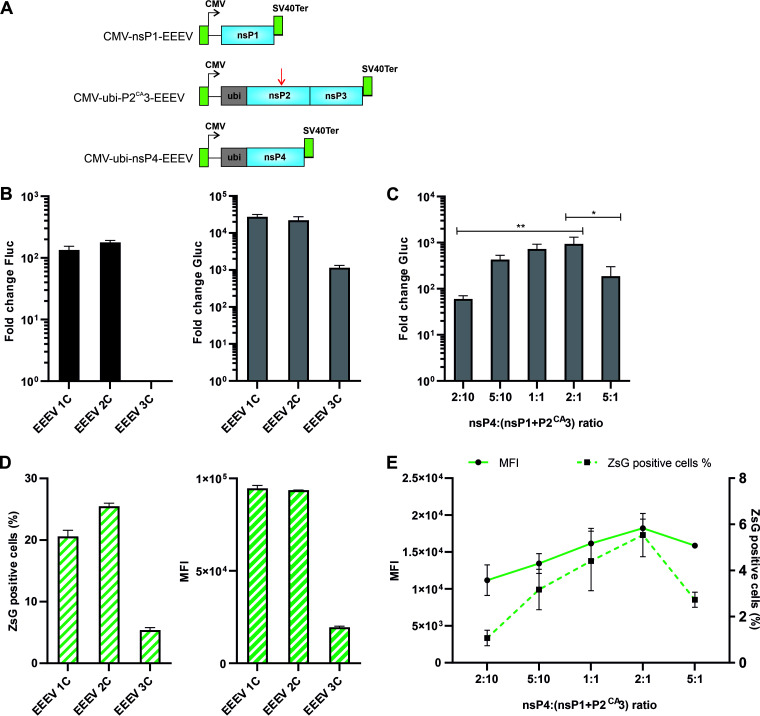
Activity of the three-component *trans-*replicase of EEEV depends on the ratio of nsP4:(nsP1+P2^CA^3) expression plasmids. (A) Schematic presentation of nsP1 (top), P2^CA^3 (middle), and nsP4 (bottom) expression constructs. Arrow indicates the position of a Cys to Ala mutation in the active site of nsP2 protease. Designations are the same as in legends of Fig. 1A and 3A. The vector backbones are not shown, and drawings are not to scale. (B) HEK293T cells in 24-well plates were cotransfected with the combinations of HSPolI-FG-EEEV and CMV-P1234-EEEV (1C), CMV-P123-EEEV + CMV-ubi-nsP4-EEEV (2C), or CMV-nsP1-EEEV + CMV-ubi-P2^CA^3-EEEV + CMV-ubi-nsP4-EEEV (3C). As a negative control, CMV-P1234^GAA^-EEEV was used. (C) HEK293T cells were cotransfected with the combinations of HSPolI-FG-EEEV, CMV-ubi-nsP1-EEEV, CMV-ubiP2^CA^3-EEEV, and CMV-ubi-nsP4-EEEV. The amounts of HSPolI-FG-EEEV, CMV-ubi-nsP1-EEEV, and CMV-ubiP2^CA^3-EEEV were kept constant, while the CMV-ubi-nsP4-EEEV was provided at a 2:10, 5:10, 1:1, 2:1, or 5:1 molar ratio in respect to CMV-nsP1-EEEV. As negative control, CMV-P1234^GAA^-EEEV was used. (B and C) Transfected cells were incubated at 37°C and lysed 18 hpt. Data represents the luciferase activity (Fluc and Gluc) from cells transfected with expression plasmid (or combination of expression plasmids) for active replicase proteins normalized to the CMV-P1234^GAA^ control cells. The value obtained for P1234^GAA^ control was taken as 1; activities lower than that observed for P1234^GAA^ are also shown as 1. The means ± SD of three independent experiments are shown. *, *P* < 0.05; **, *P* < 0.01; Student’s unpaired one-tailed *t* test. (D) HEK293T cells were cotransfected with HSPolI-FZsG-EEEV and either with CMV-P1234-EEEV (C1), CMV-P123-EEEV + CMV-ubi-nsP4-EEEV (C2), or CMV-nsP1-EEEV + CMV-ubi-P2^CA^3-EEEV + CMV-ubi-nsP4-EEEV (C3). Cells were incubated at 37°C for 18 h, collected, and analyzed with an Attune NxT acoustic focusing cytometer. The percentage of ZsGreen-positive cells from living cells (left) and MFI in arbitrary units for ZsGreen-positive cells (right) are shown. The means ± SD of three independent experiments are shown. (E) HEK293T cells were cotransfected with combinations of HSPolI-FZsG-EEEV, CMV-ubi-nsP1-EEEV, CMV-ubiP2^CA^3-EEEV, and CMV-ubi-nsP4-EEEV. The amounts of HSPolI-FZsG-EEEV, CMV-ubi-nsP1-EEEV, and CMV-ubiP2^CA^3-EEEV were kept constant, while the CMV-ubi-nsP4-EEEV was provided at a 2:10, 5:10, 1:1, 2:1, or 5:1 molar ratio in respect to CMV-nsP1-EEEV. Cells were incubated at 37°C for 18 h, collected, and analyzed with Attune NxT acoustic focusing cytometer. The percentage of ZsGreen-positive cells from living cells and MFI in arbitrary units for ZsGreen-positive cells are shown. The means ± SD of three independent experiments are shown.

The RNA replicase of SFV can be reconstructed by coexpressing the RNA template, nsP1, uncleavable P23 (P2^CA^3), and nsP4 (24). Such *trans*-replicases, hereafter designated as the “three-component replicases” (Fig. 4A), have also been constructed for SINV, CHIKV, and RRV (26). Unlike the two-component replicases that are highly active for all studied alphaviruses, the three-component replicases of different alphaviruses display remarkably divergent properties hampering their use for mix-and-match experiments (for example, in experiments using heterologous combinations of nsP1 and P2^CA^3 components). The three-component replicase of SFV had an RNA replication activity like that of the two-component replicase (24). The three-component replicase of SINV also displayed clear replication and transcription activities, albeit these were significantly lower than those observed for the corresponding two-component replicase. In contrast, low transcription activity was detected for the three-component replicase of CHIKV and no activity at all could be detected for the three-component replicase of RRV (26). Here, we constructed the three-component replicase for EEEV and performed analysis of its activities.

Activities of the one-, two-, and three-component replicases of EEEV were analyzed in HEK293T cells. The replication activity of the three-component replicase of EEEV was close to the background level, while its transcription activity was relatively high (Fig. 4B), but still approximately 23-fold lower than that of the two-component replicase. By these properties the three-component *trans*-replicase of EEEV was similar to its counterpart from SINV (26). The activity of the two-component replicase of EEEV depended on proper ratios of the nsP4 and P123 expression plasmids and suboptimal amounts of either of those components significantly reduced the viral RNA synthesis (Fig. 3). To assess if this was also the case for the three-component replicase, a titration experiment was performed where the amounts of the plasmids for the expression of the template RNA, nsP1, and P2^CA^3 were kept constant and the ratio of nsP4:(nsP1+P2^CA^3) expression plasmids varied from 2:10 to 5:1. It was found, that the transcription activities of the three-component replicase also depended on the ratio of the nsP4 expression plasmid to nsP1+P2^CA^3 expression plasmids. When the ratio of nsP4:(nsP1+P2^CA^3) expression plasmids was shifted from 2:10 to 2:1 the transcription activity increased approximately 15-fold (Fig. 4C). However, further increase of the amount of CMV-ubi-nsP4-EEEV resulted in a significant reduction of the replicases’ activities (Fig. 4C), as was the case for the two-component replicase (Fig. 3C). This data confirms that the proper ratio of the nsP4 to other components of the replicase is important for the formation and/or functioning of the RNA replicase of EEEV.

The relatively low activities of the three-component replicase of EEEV may originate from the reduction of the percentage of cells where RNA replication is initiated, lower viral RNA synthesis in such cells, or from the combination of both of these factors. To discriminate between these possibilities, we used the HSPolI-FZsG-EEEV plasmid and flow cytometry analysis. It was found that for the three-component replicase both the percentage of ZsGreen-positive cells and MFI in these cells were lower than for the two-component replicase; the difference was approximately 4.7-fold and 4.8-fold, respectively (Fig. 4D). Combined, these effects account for an approximately 22.5-fold reduction of the overall transcription activity, which is in good agreement with the 23-fold reduction observed using the HSPolI-FG-EEEV template plasmid (Fig. 4B). Thus, the lower activity of the three-component replicase of EEEV was caused both by the reduced number of cells where replication occurred and by the reduced viral RNA synthesis in these cells. This was further confirmed by analyzing the impact of the ratio of nsP4:(nsP1+P2^CA^3) expression plasmids on the transcription of the RNA template expressed from HSPolI-FZsG-EEEV. It was observed that both the percentage of ZsGreen-positive cells and MFI in these cells, was affected by the ratio of nsP4:(nsP1+P2^CA^3) expression plasmids. Again, the highest values were observed when the ratio of the nsP4:(nsP1+P2^CA^3) expression plasmids was 1:1 or 2:1. Further increase of the amount of the nsP4 expression plasmid resulted in a prominent reduction of the percentage of ZsGreen-positive cells and in a slight reduction of the MFI in these cells (Fig. 4E).

Taken together, relatively low activity of the three-component replicase of EEEV made it unsuitable for use in mix-and-match type experiments. Clearly, splitting the P123 into nsP1 and P2^CA^3 creates an additional hurdle for the formation of the active RNA replicase of EEEV. The exact reason(s) causing this effect remains unclear. One possible explanation is that this is due to the requirement of the separately expressed nsP1 and P2^CA^3 to meet and interact with each other. As the optimal stoichiometry of the ns proteins’ expression plasmids was similar for nsP4:P123 (the two-component replicase) and nsP4:(nsP1+P2^CA^3) (the three-component replicase), it is likely that splitting P123 had little or no impact on the interaction between other replicase proteins and nsP4.

### P123 of EEEV has a leading role in template RNA recognition.

**FIG 5:**
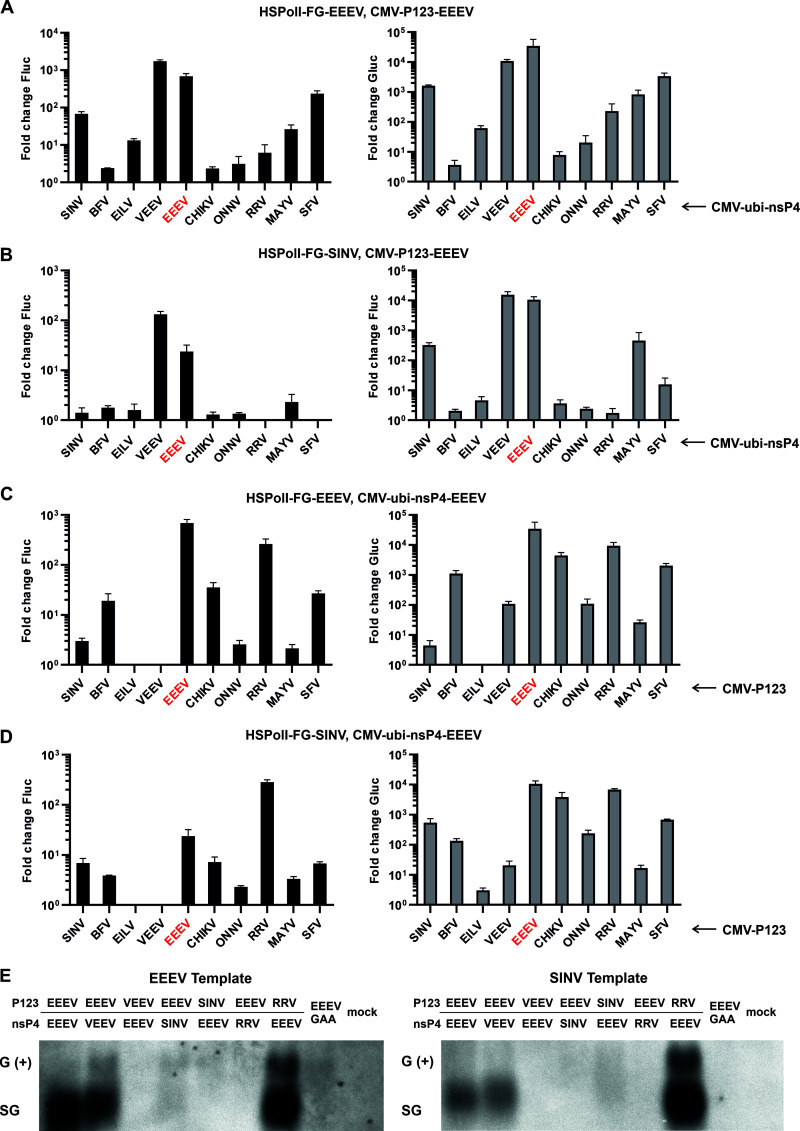
The compatibility of the P123 of EEEV with heterological nsP4s is strongly dependent on the source of the template RNA. (A) HEK293T cells in 96-well plates were cotransfected with combinations of HSPolI-FG-EEEV, CMV-P123-EEEV, and CMV-ubi-nsP4 from viruses shown on the *x* axes. (B) Experiment was performed as described for (A) except HSPolI-FG-SINV was used instead of HSPolI-FG-EEEV. (C) HEK293T cells were cotransfected with combinations of HSPolI-FG-EEEV, CMV-ubi-nsP4-EEEV, and CMV-P123 from viruses shown on *x* axes. (D) The experiment was performed as described for (C) except HSPolI-FG-SINV was used instead of HSPolI-FG-EEEV. (A–D) As negative controls, matching CMV-P1234^GAA^ plasmids were used. Transfected cells were incubated at 37°C and lysed 18 hpt. Cells transfected with plasmids originating from EILV were incubated at 28°C and lysed 48 hpt. Data represent the luciferase activity (Fluc and Gluc) from CMV-P123 + CMV-ubi-nsP4-transfected cells normalized to the paired CMV-P1234^GAA^ control cells; the value obtained for P1234^GAA^ control was taken as 1; activities lower than that observed for P1234^GAA^ are also shown as 1. The means ± SD of three independent experiments are shown. (E) HEK293T cells grown in 24-well plates were cotransfected with HSPolI-FG-EEEV (left) or HSPolI-FG-SINV (right) and combinations of P123 and nsP4 expression plasmids shown above the panel. As a negative control, CMV-P1234^GAA^-EEEV was used. At 18 hpt total RNA was extracted. Northern blot analysis was performed using 2 μg of each RNA sample and DIG-labeled RNA probe complementary to residues 42 to 390 of the sequence encoding the Gluc marker. Positions of positive-strand genomic (G [+]) and subgenomic (SG) RNAs are shown. Data from one reproducible experiment out of two is shown.

The high activity of the two-component replicase of EEEV (Fig. 3B) allowed studying the compatibility of the EEEV P123 and nsP4 proteins and template RNA with those from other alphaviruses. It was found that in the presence of the template RNA of EEEV, the combination of the P123 of EEEV with the nsP4 of VEEV outperformed other heterologous combinations and had activities similar to those of the two-component EEEV replicase (Fig. 5A). The P123 of EEEV also formed active RNA replicases with the nsP4 of SINV and several viruses from SFV complex (SFV, MAYV, and RRV). Lower activities were observed for the replicases containing the nsP4 of BFV, EILV, CHIKV, or ONNV (Fig. 5A). These results show that the P123 of EEEV has a strong preference toward the nsP4 of VEEV but no clear preference for nsP4s originating from either other outgroup or SFV complex viruses (Fig. 5A). Interestingly, there was an almost perfect correlation between the replication and transcription activities of the *trans*-replicases containing the P123 of EEEV (Fig. 5A; compare left and right panels). This suggests that the P123 of EEEV has an important role in the recognition and use of both genomic and SG promoters in the template RNAs. To analyze this further, the experiment was performed using promiscuous SINV template RNA (25) instead of the EEEV template RNA. This analysis confirmed the high compatibility of the P123 of EEEV with the nsP4 of VEEV: this heterologous combination formed an RNA replicase that had activities even higher than those of the two-component replicase of EEEV (Fig. 5B). Interestingly, however, the replication activities of all other heterologous combinations were extremely low. The same also applied to the transcription (Fig. 5B) except for the moderate activity observed for the combination of the P123 of EEEV and the nsP4 of SINV, possibly due to the match between the SINV nsP4 and the SG promoter in the template RNA of SINV. Moderate activity was also observed for the combination of the P123 of EEEV and the nsP4 of MAYV (Fig. 5B). This data clearly confirms the importance of the match between P123 and the template RNA for the formation/activity of the RNA replicases formed by the P123 of EEEV and heterologous nsP4 proteins.

The compatibility of the P123 and nsP4 components of heterologous alphaviruses is often not reciprocal (26). Therefore, the ability of the nsP4 of EEEV to form an active RNA replicase with P123s of different alphaviruses was also analyzed. Surprisingly, only background levels of replication and very modest levels of transcription activities were observed for the combination of the nsP4 of EEEV and the P123 of VEEV (Fig. 5C). At the same time, the nsP4 of EEEV formed functional replicase complexes with the P123 of BFV or viruses from the SFV complex (Fig. 5C), as previously described for the nsP4 of VEEV (26). The replication and transcription activities were highest for the RNA replicases formed with the P123s of RRV, CHIKV, SFV, and BFV. The nsP4 of EEEV failed to form an active RNA replicase with the P123 of EILV and the activities of the replicase formed with the P123 of SINV were very low (Fig. 5C). These findings are consistent with a previous observation that the P123 components of EILV and SINV do not form active RNA replicases with the nsP4s of heterologous alphaviruses (26). The ability of the nsP4 of EEEV to form an active RNA replicase with heterologous P123 components was mostly independent from the source of template RNA: the replacement of template RNA of EEEV with that of the SINV resulted only in two notable effects. Firstly, the replication and, to a smaller extent, the transcription activities of the two-component replicase of EEEV were reduced (Fig. 5D). This is in accord with the finding that the replicase proteins of EEEV prefer the matching template RNA over that of SINV (Fig. 2B). Secondly, a significant (*P* < 0.01) increase of the activities of the RNA replicase formed by the nsP4 of EEEV and the P123 of SINV was observed (Fig. 5D). This is consistent with the findings made for the P123 of EEEV (Fig. 5A and B) and likely indicates that the P123 of SINV also carries a role in the template RNA recognition and prefers the matching template RNA over that of EEEV.

To confirm that the effects revealed above were indeed caused by the differences in the viral RNA synthesis, the synthesis of positive-strand RNAs was also directly analyzed. As the analysis of all P123, nsP4, and template combinations using Northern blotting was impractical, only combinations that displayed clear and, in some cases, unexpected phenotypes were tested. Note that a faint band with mobility of genomic positive-strand RNA detected using Northern blot may not always represent a product of RNA replicase activity as the primary noncapped RNA transcript made by cellular RNA polymerase I has identical mobility; thus, the presence of RNA with mobility of SG RNA serves as a more reliable indicator of activity of analyzed combinations. As expected, the two-component replicase of EEEV was highly active on the matching RNA template while its activity on the SINV template was lower; the same also applied for replicase formed by P123 of EEEV and nsP4 of VEEV (Fig. 5E). In contrast, the activity of the replicase formed by P123 of VEEV and nsP4 of EEEV was below the detection limit for the analysis on both template RNAs (Fig. 5E). Replicase consisting of the P123 of EEEV and the nsP4 of SINV had detectable activity on EEEV template RNA but not on SINV RNA; the situation was reversed for the replicase consisting of the P123 of SINV and the nsP4 of EEEV (Fig. 5E). The activity of the replicase consisting of the P123 of EEEV and the nsP4 of RRV was below the detection limit for the Northern blot assay while the activity of the reciprocal combination was very high and did not depend on the source of the template RNA (Fig. 5E). All these observations are consistent with the data obtained by analysis of the activities of expressed marker proteins (Fig. 5A–D), clearly confirming the validity of the conclusions presented above. In addition, it was found that the assay based on the measurement of marker protein activities was more sensitive and reliable, allowing the detection and comparison of activities of replicases with low activities, unlike the Northern blot assay.

Taken together, it was found that the ability of the EEEV P123 to form a functional RNA replicase with heterologous nsP4s was more pronounced in the presence of the template RNA of EEEV. In contrast, the nsP4 of EEEV displayed lesser preference for template RNA. At the same time, the preference toward its own template RNA was also observed for the P123 of SINV.

### Constructs expressing template RNAs of CHIKV or EEEV can be used as biosensors.

Minigenomes of SINV and CHIKV, harboring sequences encoding for a fluorescent marker under the SG promoter and bearing similarity to the template RNAs used in this study have been used for detection of alphavirus infection in cultivated mosquito cells. These sensors could activate reporter expression upon infection with corresponding or related alphaviruses; however, the activation achieved in the transient expression system was less than 10-fold (31). This modest activation may reflect a suboptimal design of the template RNA and/or limitations specific to the mosquito cells as we have previously observed >10,000-fold activation of the Gluc reporter when HSPolI-FG-CHIKV transfected human cells were infected with CHIKV (30). Similar high-level activation was also observed in our *trans*-replicase experiments (Fig. 1B) indicating the possibility that the alphavirus template RNAs, transcribed in human cells from plasmid DNA, have the potential to be used as detection and enumeration tools for alphaviruses, allowing modern direct fluorescence and luminescence technologies to be used for unmodified wild-type viruses. Furthermore, given the revealed cross-utilization of template RNAs by replicases of different alphaviruses it was envisioned that cells transfected with plasmids encoding such template RNAs can be used as biosensors for identification of specific alphaviruses or groups of alphaviruses. These possibilities were analyzed using HEK293T cells transfected with HSPolI-FZsG-EEEV or HSPolI-FZsG-CHIKV template RNA expression plasmids.

**FIG 6:**
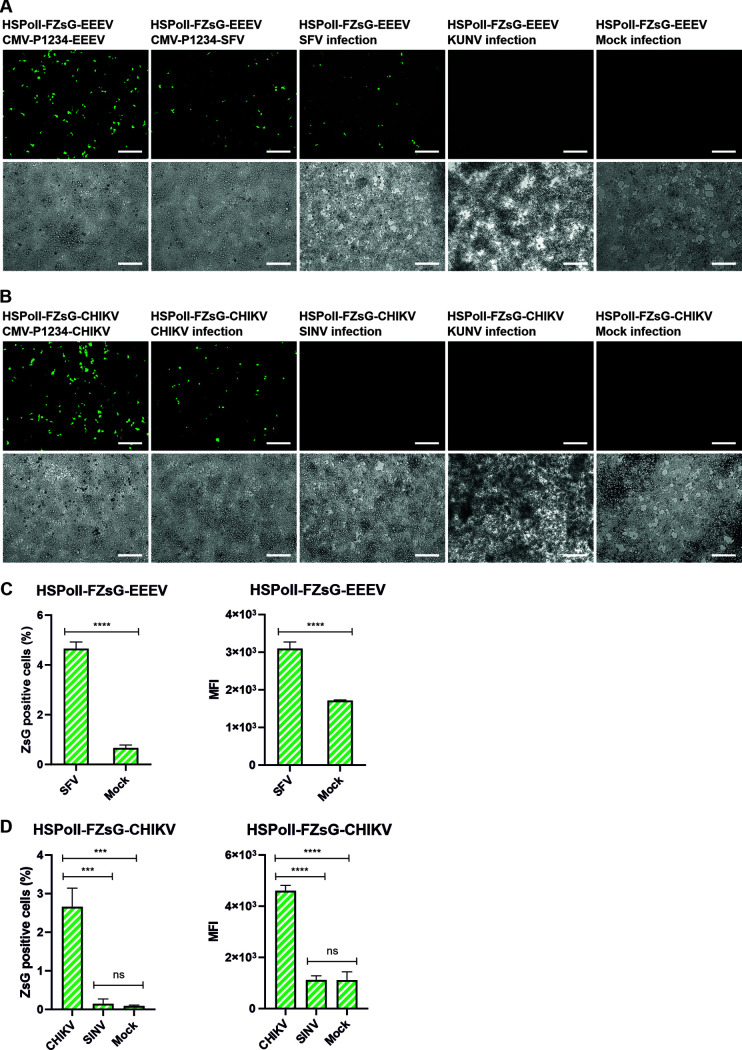
EEEV and CHIKV template RNAs work as biosensors of Old World alphavirus infection. (A) HEK293T cells were either cotransfected with HSPolI-FZsG-EEEV and CMV-P1234-EEEV or CMV-P1234-SFV, or transfected with HSPolI-FZsG-EEEV, and infected 24 hpt with SFV at MOI 10, with KUNV at MOI 0.1 or mock infected. (B) HEK293T cells were either cotransfected with HSPolI-FZsG-CHIKV and CMV-P1234-CHIKV or transfected with HSPolI-FZsG-CHIKV and infected 24 hpt with CHIKV or SINV at MOI 10, with KUNV at MOI 0.1 or mock infected. (A and B) At 18 hpt or 24 hpi (168 hpi for KUNV) cells were observed using the EVOS M7000 imaging system for expression of ZsGreen reporter (top panels) and the presence of CPE (bottom panels). Representative images from three reproducible independent experiments are shown. Scale bars: 200 μm (C) HEK293T cells were transfected with HSPolI-FZsG-EEEV and infected at 24 hpt with SFV at MOI 10 or mock infected. (D) HEK293T cells were transfected with HSPolI-FZsG-CHIKV and infected at 24 hpt with CHIKV or SINV at MOI 10 or mock infected. (C and D). At 24 hpi, cells were collected, fixed, and analyzed with the Guava easyCyte HT system. The percentage of green cells (left panels) and MFI in arbitrary units in fluorescent cells (right panels) are shown. The means ± SD of three independent experiments are shown. ns, not significant; **, *P* < 0.01; ***, *P* < 0.001; ****, *P* < 0.0001; Student’s unpaired one-tailed *t* test.

It was observed that the cotransfection of HEK293T cells with HSPolI-FZsG-EEEV and CMV-P1234-EEEV resulted in the appearance of numerous ZsGreen-expressing cells (Fig. 6A, Movie S1). The cotransfection of HSPolI-FZsG-EEEV and CMV-P1234-SFV, a less potent activator of transcription from the EEEV template RNA (Fig. 2A), predictably resulted in a somewhat less prominent but clear ZsGreen expression (Fig. 6A). Importantly, clear ZsGreen expression was also observed when HSPolI-FZsG-EEEV-transfected cells were infected with SFV (Fig. 6A, Movie S2). It was found that compared to mock infection, SFV infection resulted in an approximately 7-fold increase of percentage of fluorescent cells as well as a nearly 2-fold and highly significant increase of MFI in these cells (Fig. 6C). No ZsGreen expression was observed if the cells were infected with flavivirus Kunjin virus (KUNV) (Fig. 6A). Thus, replication and transcription of the EEEV template RNA could be activated by infection of SFV and presumably by any alphavirus, the replicase of which is capable of cross utilizing the template RNA of EEEV (Fig. 2A). The experiment performed using CHIKV templates confirmed this assumption. In HSPolI-FZsG-CHIKV-transfected cells the ZsGreen expression could be activated by coexpression of CHIKV P1234 and by infection with CHIKV (Fig. 6B; Movies S3 and S4) but not by infection with SINV (Fig. 6B; Movie S5), the replicase of which is unable to activate replication of the template RNA of CHIKV (25). Consistently, it was observed that compared to mock infection, CHIKV infection of HSPolI-FZsG-CHIKV-transfected cells resulted in an approximately 33-fold increase of percentage of fluorescent cells as well as a nearly 4-fold increase of MFI in these cells. Neither of these effects was observed upon infection with SINV (Fig. 6D).

**FIG 7:**
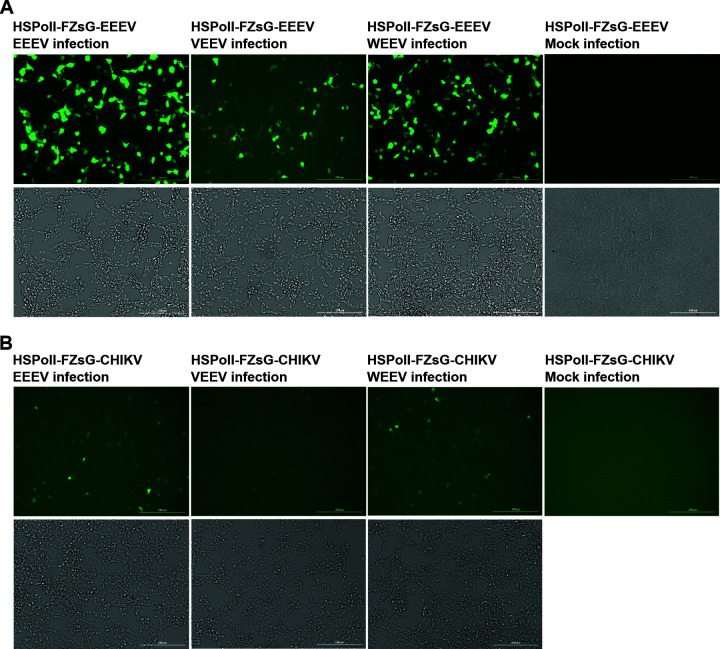
HSPolI-FZsG-EEEV can be used as a highly sensitive biosensor for New World alphavirus infection. HEK293T cells were transfected with HSPolI-FZsG-EEEV (A) or HSPolI-FZsG-CHIKV (B). After overnight incubation, cells were infected with EEEV, VEEV, or WEEV at MOI 5, or mock infected. At 24 hpi cells were fixed in 10% neutral buffered formalin. Images were taken using a BioTek Cytation1 digital fluorescence microscope; scale bars: 200 μm.

For assessment of the ability of different New World alphaviruses to activate EEEV and CHIKV templates, cells transfected with HSPolI-FZsG-EEEV or HSPolI-FZsG-CHIKV were infected with EEEV, VEEV, or WEEV. EEEV template RNA was activated with high efficiency by EEEV and by WEEV infection, and to a lesser but genuine extent by VEEV infection (Fig. 7A). For VEEV-infected cells, the number of fluorescent cells and fluorescence intensity were lower than for other New World alphaviruses (Fig. 7A). This is consistent with the observation that VEEV replicase uses the EEEV template relatively inefficiently (Fig. 2A). For cells transfected with the CHIKV template RNA expression plasmid, all three New World alphaviruses activated ZsGreen expression to a much lesser extent than in the cells that harbored the EEEV template. However, the order of relative fluorescence intensity upon New World alphavirus infection was the same: EEEV followed by WEEV, with VEEV being the weakest activator (Fig. 7B). Again, this data is consistent with the moderate ability of EEEV replicase to use CHIKV template RNA (Fig. 2B) as well as with previous observation that VEEV replicase has a very limited ability to use CHIKV template RNA (25). Taken together, our data confirms that the designed EEEV RNA template can be used for the detection of New World as well as Old World alphavirus infections. The replicase of WEEV appears to have similar template requirements to those of EEEV replicase; however, the construction of the WEEV *trans*-replicase system was outside the scope of this study.

## DISCUSSION

The *trans*-replicases of alphaviruses represent efficient and reliable tools for studying the interactions between template RNA and replicase proteins, formation of RCs, and interactions between ns proteins, as well as interactions between ns proteins and host proteins (14, 20, 32–34). Here, we developed and used several variants of *trans*-replicases for EEEV. Both one- and two-component EEEV *trans*-replicases were highly active, providing an opportunity to apply this system for studying EEEV RNA replication. In the future, the system could also be used to study the impact of mutations on EEEV RNA replication, as decrease in replicase activities would be easily observable. Here, we have demonstrated that EEEV *trans*-replicases can be used for analysis of the interactions between replicase components from different alphaviruses.

The high activity of the EEEV *trans*-replicase in HEK293T cells contrasts to the much lower activity of VEEV *trans*-replicase (Fig. 1B). Data from biosensor experiments (Fig. 7) indicates that the relatively weak ability of P1234 of VEEV to use heterologous templates observed previously (25) and in this study (Fig. 2A) is not an artifact caused by expression of replicase proteins from plasmid vectors. Instead, the replicase of VEEV seems to have a reduced ability to replicate heterologous templates provided in *trans*. Though the ability of VEEV replicase to transcribe the matching template was also lower than that of the EEEV replicase, the transcription nevertheless occurred at a high level (Fig. 1B). This is consistent with the report that both EEEV and VEEV replicon vectors can produce packaged virus-replicon particles in BHK-21 cells cotransfected with the transcripts of corresponding replicon and helper RNAs (35). The experiments with two-component replicases revealed that the low replication activity of VEEV replicase is not due to the nsP4 component, as a replicase consisting of the P123 of EEEV and the nsP4 of VEEV has activities similar to, or even higher, than the highly active EEEV two-component replicase (Fig. 5A and B). This suggests that the relatively low activity of VEEV *trans*-replicase is associated with the P123 component. Interestingly, increasing the amount of nsP4 expression plasmid to above a 1:1 ratio with P123 expression plasmid resulted in a reduction of EEEV RNA replication. During infection, nsP4 is generally produced via translational read-through of a stop codon at the end of the nsP3 region; in addition, nsP4 is also degraded by proteasomes. These two mechanisms ensure the optimal ratio of different nsPs in alphavirus-infected cells. A very recent study revealed that in the replicase core of an alphavirus, nsP1 and nsP4 are present in a 12:1 molar ratio (18). The fixed 12:1 ratio explains why the deficit of nsP4 hampers RC formation; however, it cannot explain why the excess of nsP4 has a similar effect. One possible explanation is provided in the same study, where it was revealed that nsP4 not involved in viral RNA synthesis is also present in a complex with 12 molecules of nsP1 and one molecule of nsP2. Thus, it is possible that an excess of nsP4 over nsP1 and the template RNA causes excessive formation of such inactive complexes at the expense of true RCs. Alternatively, an excess of nsP4 may trigger cellular defense mechanisms and/or interfere with important cellular processes, resulting in the inhibition of RNA replication. Interestingly, however, activities of two-component *trans*-replicases of CHIKV, BFV, SINV, VEEV, and EILV were all increased by ratios of nsP4:P123 expression plasmid above 1:1 (26), raising the question: is the bell-shape dependence of the replication activity from the nsP4:P123 expression plasmid ratio a specific property of EEEV? As the proper folding of nsP4 depends on its interaction with nsP1 (18), it seems plausible that the alphaviruses likely differ from each other by efficiency of nsP1:nsP4 interactions and/or by the conformation which nsP4 acquires prior to the interaction with nsP1. It is likely that the extremely high activities of one- and two-component *trans*-replicases of EEEV may reflect the fact that its nsP4 is stable enough to compensate for the extra time required to associate with the template RNA provided in *trans* and, for the two-component system, also with nsP1. For other alphaviruses these interactions may be more time critical and therefore compensated by the excess of nsP4.

The template RNA of EEEV was found to be quite universal, as it could be replicated and transcribed by the replicases of outgroup and SFV complex viruses (Fig. 2A). The same has been previously observed for VEEV template RNA and, with small variations, for template RNAs of other outgroup alphaviruses (25). Similar to replicases of other outgroup alphaviruses (25), the replicase of EEEV was efficient only with template RNAs belonging to outgroup alphaviruses (Fig. 2B).

We have shown previously that functional alphavirus replicases can be reconstructed from heterologous P123 and nsP4 components (26). Here, we found that nsP4 of EEEV was capable of forming active replicases with P123 components from viruses belonging to the SFV complex (Fig. 5C and D, Fig. 6). This pattern was nearly identical to that observed for nsP4 of VEEV (26). Furthermore, like nsP4 of VEEV, nsP4 of EEEV was compatible with P123 of BFV but was very poorly compatible with P123 of SINV and not at all with P123 of EILV (Fig. 5C). However, despite these similarities between EEEV and VEEV, the complexes formed by nsP4 of EEEV and P123 of VEEV displayed very low transcription and no detectable replication activity (Fig. 5C and D, Fig. 6).

In the presence of the matching template RNA, P123 of EEEV formed active replicases with nsP4 of VEEV, SINV, or SFV (Fig. 5A). Surprisingly, when the template originated from SINV, the only highly active heterologous combination was P123 of EEEV + nsP4 of VEEV (Fig. 5B). Furthermore, it was noted that the heterologous replicase formed by P123 of EEEV and nsP4 of SINV was more efficient on the EEEV RNA template (compare Fig. 5A and B) while the replicase formed by P123 of SINV and nsP4 of EEEV preferred the SINV template RNA (compare Fig. 5C and D). Combined, this data suggests that the P123 components of EEEV and SINV have a crucial role in template RNA recognition. Thus, experiments performed using different alphaviruses indicate a leading role of either P123 (as in this study) or nsP4 (26) in template RNA recognition. We propose that all alphaviruses (with the possible exception of those infecting fish) have a common mechanism of template RNA recognition and utilization that involves recognition of template RNA by the P123 component of the replicase. In addition, alphaviruses from the SFV complex have acquired another step, in which nsP4 contributes to the specificity of template recognition allowing differentiation between templates of closely related viruses (26). The additional mechanism may be due to highly conserved RNA secondary structures in the 5′ regions of the genomes of these viruses and is possibly responsible for the fact that the replicases of outgroup viruses cannot efficiently use the templates of viruses from the SFV complex (25). However, the possibility that viruses from the SFV complex use nsP4 alone, while outgroup viruses use P123, for template RNA recognition cannot be excluded.

Constructs expressing CHIKV and EEEV template RNAs were found to function as potential biosensors for alphavirus infection. There was a clear correlation between the ability of the viral replicase to replicate and transcribe specific template RNA and the ability of the corresponding virus to trigger marker expression from a sensor based on such template RNA (Fig. 6 and 7). This opens possibilities for the rational design of biosensors with desired properties. Such biosensors can be used for virus quantitation by focus-forming assay or limiting dilution using automated fluorescence microscopy, and by limiting dilution using luminescence readers. Biosensors could be used for virus enumeration in real time, without fixation and staining protocols, and in the case of fluorescence facilitating recovery of virus from wells where required. This would be extremely useful in biosafety level 3 laboratories, reducing the manipulation of virus while allowing repeated measurements of the same sample. Stable cell lines harboring biosensors would also be useful for different screening approaches as they allow the use of nonmodified natural virus isolates instead of clone-derived marker viruses. The fact that one and the same biosensor can be activated by several alphaviruses allows application of these approaches to many viruses eliminating the need for generating large numbers of different marker viruses.

Improved mechanistic understanding of RNA replicase functions is needed for bettering understanding of the pathogenicity of EEEV and its host range, and thus for improving the efficacy of medical treatments and prophylaxis. Understanding the precise mechanisms of EEEV infection can also contribute to continued research on vaccine candidates and antivirals. It also opens possibilities for the rational design of reporter-carrying models based on mammalian and possibly mosquito vectors that may have multiple uses for advanced studies of virus infection *in vivo.* Such models would be especially important for studying those stages of infection that are poorly understood due to low quantities of virus (and thus limited sensitivity), including the establishment of infection and potentially the long-term persistence of alphaviruses in mammalian hosts and mosquito vectors.

## MATERIALS AND METHODS

### Cells and viruses.

HEK293T human embryonic kidney cells (ATCC CRL-3216) were maintained in Dulbecco’s modified Eagle medium (DMEM) with 2 mM l-glutamine and 10% fetal bovine serum (FBS) at 37°C in a 5% CO_2_ humidified atmosphere. BHK-21 baby hamster kidney cells (ATCC CCL-10) were maintained in Glasgow’s modified Eagle’s medium (GMEM) supplemented with 10% FBS, 10% tryptose phosphate broth, 20 mM HEPES, and 1 mM l-glutamine at 37°C in a 5% CO_2_ humidified atmosphere. U2OS human bone osteosarcoma cells (ATCC HTB-96) were maintained in Iscove’s modified Dulbecco’s medium (Gibco) containing 10% FBS and 2 mM l-glutamine at 37°C in a 5% CO_2_ humidified atmosphere. U2OS-derived double-null ΔΔG3BP1/2-KO cells (36) were maintained in the same medium as wild-type U2OS cells. Media for HEK293T, BHK-21, U2OS, and U2OS ΔΔ cells was supplemented with 100 IU/mL penicillin and 0.1 mg/mL streptomycin. Vero African green monkey cells (European Collection of Animal Cell Cultures, UK) were maintained in DMEM with 50 IU/mL penicillin, 50 μg/mL streptomycin, 2 mM l-glutamine, and 10% FBS at 37°C in a 5% CO_2_ humidified atmosphere. For virus infections at biosafety level 3, Vero cells were maintained in Leibovitz L-15 medium supplemented with 50 IU/mL penicillin, 50 μg/mL streptomycin, 2 mM l-glutamine, and 2% FBS at 37°C in a humidified atmosphere without added CO_2_.

### Viruses.

SFV (isolate SFV6), SINV (isolate Toto1101), and CHIKV (isolate LR2006OPY1 of East/Central/South African genotype) were rescued from corresponding infectious cDNA clones (37–39). Infectious cDNA clone of KUNV (isolate FLSDX, GenBank: AY274504) (40) was assembled in pCCI-BAC vector using synthetic DNA fragments (Genscript, USA). SFV, SINV, and CHIKV were rescued, propagated, and plaque-titrated in BHK-21 cells. KUNV was rescued, propagated, and plaque-titrated in Vero cells. Stocks of EEEV (PE-6), WEEV (Fleming), and VEEV (TrD) were prepared in suckling mice as described previously (41–43) and the titers were determined by standard plaque assay methods in Vero cells utilizing a carboxymethyl cellulose overlay.

### Plasmids.

Human RNA polymerase I promoter-based plasmids for the production of replication-competent RNA templates designated HSPolI-FG-CHIKV, HSPolI-FG-ONNV, HSPolI-FG-RRV, HSPolI-FG-SFV, HSPolI-FG-MAYV, HSPolI-FG-SINV, HSPolI-FG-BFV, HSPolI-FG-VEEV, HSPolI-FG-EILV, where FG stands for Fluc and Gluc markers, have been previously described (20, 25, 26). The plasmid for the expression of the RNA template of EEEV had the same design and was assembled from synthetic DNA fragments (Genscript, USA) and was designated HSPolI-FG-EEEV. To obtain an RNA template plasmid containing the ZsGreen marker, the sequence encoding Gluc in the HSPolI-FG-EEEV was substituted with the sequence encoding ZsGreen using restriction-based cloning; the obtained plasmid was designated HSPolI-FZsG-EEEV. HSPolI-FZsG-CHIKV, a plasmid of similar design encoding for template RNA of CHIKV has been previously described (26).

Plasmids encoding P1234 of selected alphavirus and designated CMV-P1234-CHIKV, CMV-P1234-ONNV, CMV-P1234-RRV, CMV-P1234-SFV, CMV-P1234-MAYV, CMV-P1234-SINV, CMV-P1234-BFV, CMV-P1234-VEEV, and CMV-P1234-EILV, as well as their variants encoding polymerase-negative variant P1234^GAA^ (CMV-P1234^GAA^-CHIKV and so on), have been previously described (20, 25, 30). The plasmid for expression of P1234 of EEEV had the same design as other plasmids; the sequence encoding for P1234 of EEEV was assembled from synthetic DNAs (Genscript, USA). Its polymerase-negative variant was obtained using site-directed mutagenesis and subcloning procedures. The plasmids were designated CMV-EEEV-P1234 and CMV-P1234^GAA^-EEEV, respectively.

Plasmids encoding P123 of alphaviruses listed above, designated CMV-P123-CHIKV and so on, have been previously described (26). Plasmids encoding ubi-nsP4 of alphaviruses listed above, designated CMV-ubi-nsP4-CHIKV and so on, have also been previously described (26). The plasmid for the expression of P123 of EEEV had a similar design and was constructed using CMV-EEEV-P1234 plasmid and PCR-amplified fragments; the obtained construct was designated CMV-P123-EEEV. The plasmid for the expression of ubiquitin-fused nsP4 of EEEV was constructed using synthetic DNAs (Genscript, USA) and fragments of CMV-EEEV-P1234 plasmid. The obtained construct was designated CMV-ubi-nsP4-EEEV.

The plasmid expressing nsP1 of EEEV was constructed using PCR-amplified fragments and the CMV-EEEV-P1234 plasmid and was designated CMV-nsP1-EEEV. To construct the plasmid designated as CMV-P2^CA^3-EEEV, the nsP1 coding region in CMV-P123-EEEV was replaced by a region encoding ubiquitin, and the codon corresponding to the catalytic Cys residue in the active site of nsP2 protease was replaced with the codon of Ala using PCR-based mutagenesis. Sequences of all plasmids containing fragments of EEEV cDNA were verified using Sanger sequences and are provided in the supplemental Sequences file.

### *Trans*-replication assay.

For HEK293T cells, the *trans*-replication assay was carried out using a 96-well or a 24-well plate format, as previously described (25, 26). Briefly, for 96-well plates, approximately 35,000 cells per well were cotransfected with 50 ng of plasmids encoding template RNA and 50 ng of plasmids encoding P1234. For two-component replicases, 50 ng of P123-encoding plasmids were combined with an equimolar amount of nsP4 expression plasmids. To determine the dependence of RNA synthesis efficiency from the ratio of nsP4 to P123, molar ratios of nsP4- and P123-encoding plasmids of 1:10, 2:10, 4:10, 6:1, 8:10, 1:1, 2:1, 4:1, 6:1, and 8:1 were used. As negative controls, CMV-P1234^GAA^ constructs were used. To increase sensitivity, the three-component *trans*-replicase assays were conducted in a 24-well format, where 250 ng of plasmids encoding template RNA and 190 ng of the nsP4-encoding plasmid were combined with equimolar amounts of nsP1- and ubi-P2^CA^3-encoding plasmids. In the experiments aiming the determination of the dependence of RNA synthesis efficiency of the three-component *trans*-replicase from the ratio of nsP4 to nsP1, the cells were transfected as described above, except that the amount of the nsP4-encoding plasmid was 38 ng (2:10 ratio), 95 ng (5:10 ratio), 190 ng (1:1 ratio), 380 ng (2:1 ration), or 950 ng (5:1 ratio). As negative controls, CMV-P1234^GAA^ constructs were used instead of plasmids encoding for P1234, P123+nP4, or nsP1+P2^CA^3+nsP4. Transfections were performed using Lipofectamine LTX with PLUS reagent (Thermo Fisher Scientific) according to the manufacturer’s instructions. Transfected cells were incubated at 37°C for 18 h; as an exception, the cells transfected with EILV P123 and nsP4 expression plasmids were incubated at 28°C for 48 h.

All transfections were performed in triplicate, and experiments were repeated at least twice. After incubation, cells were lysed, and Fluc and Gluc activities were measured using the Dual-Luciferase reporter assay (Promega). Fluc and Gluc activities measured for cells transfected using plasmids expressing active replicases were normalized to those obtained for the corresponding control cells.

U2OS or U2OS ΔΔ cells grown on 12-well plates were cotransfected with 1 μg of HSPolI-FG-EEEV and 1 μg of CMV-P1234-EEEV using Lipofectamine LTX with PLUS reagent (Thermo Fisher Scientific) according to the manufacturer’s instructions. In this experiment, plasmid combinations of HSPolI-FG-CHIKV and CMV-P1234-CHIKV or HSPolI-FG-VEEV and CMV-P1234-VEEV were used as controls. All transfections were performed in triplicate and experiments were repeated at least twice. After incubation, cells were lysed, and Fluc and Gluc activities were measured using the Dual-Luciferase reporter assay (Promega). For each virus, Fluc and Gluc activities measured for the U2OS ΔΔ cells were compared with those obtained for wild-type U2OS cells.

### Flow-cytometry assay.

For the two-component *trans*-replicase of EEEV, approximately 300,000 HEK293T cells were grown in a well of 24-well plates and cotransfected with 250 ng of HSPolI-FZsG-EEEV and 250 ng of CMV-P123-EEEV combined with CMV-ubi-nsP4-EEEV using the following molar ratios of nsP4- and P123-encoding plasmids: 1:10, 2:10, 4:10, 6:1, 8:10, 1:1, 2:1, 4:1, 6:1, and 8:1. For the three-component replicase, HEK293T cells in 24-well plates were cotransfected with 250 ng of HSPolI-FZsG-EEEV, 190 ng of CMV-ubi-nsP4-EEEV and equimolar amounts of CMV-nsP1-EEEV and CMV-ubi-P2^CA^3-EEEV. In the experiments that aimed to determine the dependence of the percentage of cells with RNA replication and transcription activity of the EEEV three-component *trans*-replicase in such cells from the ratio of nsP4 to nsP1, the cells were transfected as described above, except that the amount of the nsP4-encoding plasmid was 38 ng (2:10 ratio), 95 ng (5:10 ratio), 190 ng (1:1 ratio), 380 ng (2:1 ration), or 950 ng (5:1 ratio). Transfections were performed using Lipofectamine LTX with PLUS reagent. Cells were collected in 500 μL PBS at 18 hours posttransfection (hpt) and analyzed with the Attune NxT acoustic focusing cytometer. At least 30,000 events were recorded for each sample. The obtained data were analyzed using Attune NxT software to determine the percentage of living cells, the percentage of cells positive for ZsGreen marker, and the MFI of ZsGreen (in arbitrary units) in marker-positive cells. The experiment was repeated three times.

### Northern blotting.

Northern blotting was performed as previously described (26). Briefly, approximately 300,000 HEK293T cells grown in 24-well plates were cotransfected with 0.5 μg of template RNA expression plasmid and 0.5 μg of P123 expression plasmid combined with an equimolar amount of nsP4 expression plasmid using Lipofectamine LTX with PLUS reagent; control cells were mock transfected. At 18 hpt, total RNA was extracted using TRIzol reagent (Life Technologies). Then, 2 μg of total RNA were denatured for 10 min at 70°C in 2× RNA loading dye (Thermo Fisher Scientific), cooled on ice, and separated on a denaturing gel (1% agarose, 6% formaldehyde) using 1× morpholinepropanesulfonic acid (MOPS) buffer. RNA was transferred to a Hybond-N+ filter (GE Healthcare) and fixed using a UV Stratalinker 1800 (Stratagene). A digoxigenin (DIG)-labeled RNA probe complementary to residues 42 to 390 of the sequence encoding the Gluc marker was used to detect positive-strand RNAs. Filters were hybridized overnight; blots were washed and developed according to the manufacturer’s (Roche) protocols.

### Biosensor assays.

For biosensor assays, HEK293T cells were seeded at 200,000 cells per well of a 24-well cell culture plate. Cells were grown for 24 h, after which they were transfected with 0.5 μg of HSPolI-FZsG-EEEV or HSPolI-FZsG-CHIKV using Lipofectamine LTX and PLUS reagents (Thermo Fisher Scientific); control cells were mock transfected. After overnight incubation, cultures were infected with SFV, SINV, or CHIKV at MOI 10, with KUNV at MOI 0.1 or mock infected. At 24 h postinfection (hpi) (168 hpi for KUNV), cells were observed using the EVOS M7000 imaging system (Thermo Fisher Scientific) to assess the presence of CPE and the production of ZsGreen reporter. CPE development and ZsGreen expression in cells transfected using HSPolI-FZsG-EEEV or HSPolI-FZsG-CHIKV and infected with SFV, SINV, or CHIKV were also recorded for 24 h with images taken using the EVOS M7000 imaging system every 30 min. Microscopy image adjustments were made with the Celleste 5 software (Thermo Fisher Scientific), organized to appropriate time-lapse panels in CorelDraw 2020, and finalized as animations with the ZEN 3.3 blue edition software (Carl Zeiss Microscopy).

To determine the percentage of cells positive for ZsGreen marker and the MFI in ZsGreen positive-cells, the HEK293T cells were transfected and infected as described above. At 24 hpi, the cells were treated with 120 μL of trypsin and fixed with 120 μL of 8% formaldehyde in Dulbecco’s Phosphate-Buffered Saline so that the final concentration of formaldehyde solution was 4%. Fixed cells were analyzed with Guava easyCyte HT system; 10,000 events were recorded for each sample. The obtained data were analyzed using Guava InCyte software.

Biosensor assays with EEEV, VEEV, and WEEV were performed as described above with following modifications. HEK293T cells were transfected with HSPolI-FZsG-EEEV or HSPolI-FZsG-CHIKV using *Trans*IT-LT1 transfection reagents (Mirus Bio) according to manufacturer’s instructions; control cells were mock-transfected. After overnight incubation, cultures were infected with EEEV, VEEV, or WEEV at MOI 5, or mock infected. At 24 hpi, cultures were observed for CPE development. Cultures were fixed overnight in 10% neutral buffered formalin and removed from the high-containment laboratory after formaldehyde fumigation. Cell monolayers were visualized using a BioTek Cytation1 digital fluorescence microscope.

### Statistical analysis.

Statistical analysis was performed using GraphPad Prism 8.2.0 software. Data were analyzed using Student’s unpaired one-tailed *t* test. *P* values of ≤0.05 (*), ≤0.01 (**), ≤0.001 (***), and ≤0.0001 (****) were used to represent degrees of significance. Each experiment was repeated to gain a minimum of three independent biological repeats.

### Data availability.

All data used to reach the conclusions in this paper and any data required to replicate the study findings is presented in the manuscript and supplemental Raw Data and Raw Images files. Sequence of plasmids encoding for EEEV proteins, polyproteins, and template RNAs are provided in the Sequences file. All plasmids used in the study are available from authors without restrictions.
