# Supplementary material for: Activity, Template Preference, and Compatibility of Components of RNA Replicase of Eastern Equine Encephalitis Virus
Source: J Virol. 2022 Dec 19;97(1):e01368-22. doi: 10.1128/jvi.01368-22 (PMC9888243; doi:10.1128/jvi.01368-22)

Supplements:

Movie S1: Co-transfection of HEK293T cells with HSPolI-FZsG-EEEV and CMV-P1234-EEEV

Movie S2: Infection of HEK293T cells transfected with HSPolI-FZsG-EEEV with SFV

Movie S3: Co-transfection of HEK293T cells with HSPolI-FZsG-CHIKV and CMV-P1234-CHIKV

Movie S4: Infection of HEK293T cells transfected with HSPolI-FZsG-CHIKV with CHIKV

Movie S5: Infection of HEK293T cells transfected with HSPolI-FZsG-CHIKV with SINV

Raw Data: file containing raw data (measurement of Fluc and Gluc activities, percentage of ZsGreen positive cells and MFI) of trans-replicase assays

Sequences: file containing sequence of HSPolI-FG-EEEV, HSPolI-FZsG-EEEV, CMV-P1234-EEEV, CMV-P1234^{GAA}-EEEV, CMV-P123-EEEV, CMV-P2^{CA}3-EEEV, CMV-nsP1-EEEV, and CMV-ubi-nsP4-EEEV

Original caption of image (Figure 5E, left panel)

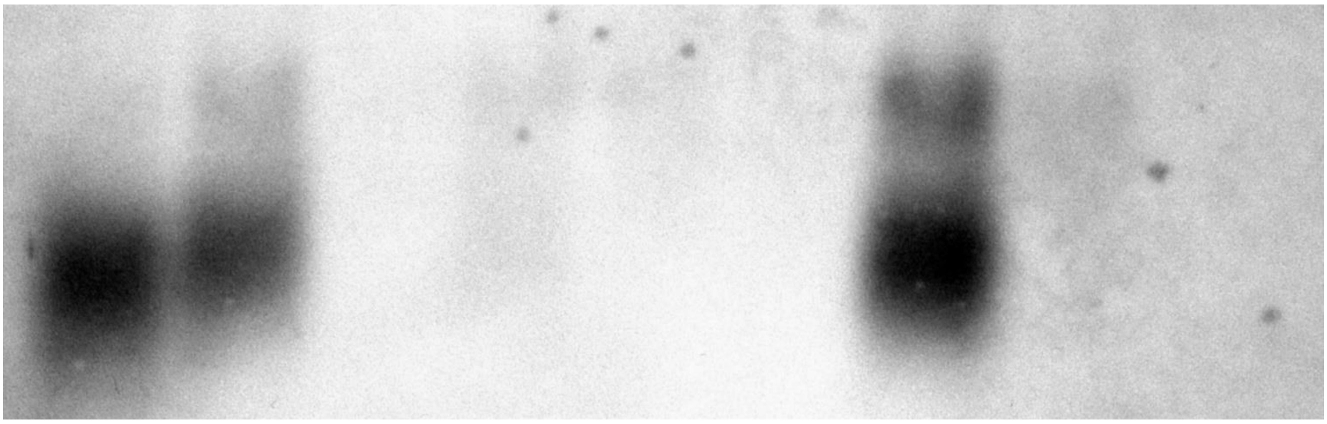

Original caption of image (Figure 5E, right panel)

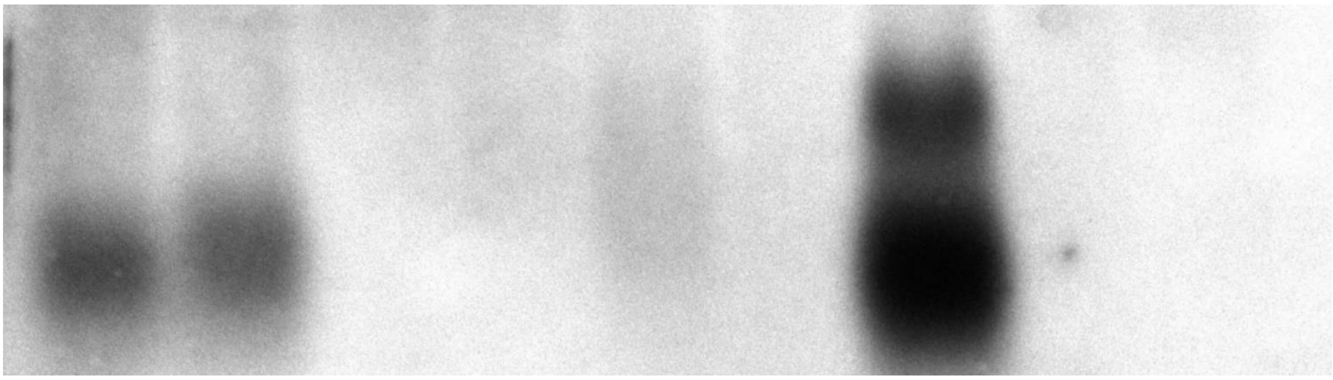

Supplement: Supplemental file 1 — Supplemental material legends and original captures. Download jvi.01368-22-s0001.pdf, PDF file, 0.2 MB [file jvi.01368-22-s0001.pdf]
